# Identification of D842V mutation in gastrointestinal stromal tumors based on CT radiomics: a multi-center study

**DOI:** 10.1186/s40644-024-00815-3

**Published:** 2024-12-20

**Authors:** Zhenhui Xie, Qingwei Zhang, Ranying Zhang, Yuxuan Zhao, Wang Zhang, Yang Song, Dexin Yu, Jiang Lin, Xiaobo Li, Shiteng Suo, Yan Zhou

**Affiliations:** 1 Department of Radiology, Renji Hospital, School of Medicine, Shanghai Jiao Tong University, Pujian Road 160, Pudong District, 200127 Shanghai, China; 2 Division of Gastroenterology and Hepatology, Key Laboratory of Gastroenterology and Hepatology, Ministry of Health, Renji Hospital, School of Medicine, Shanghai Jiao Tong University, Shanghai Institute of Digestive Disease, Pujian Road 160, Pudong District, 200127 Shanghai, China; 3 Department of Radiology, Zhongshan Hospital, Fudan University, and Shanghai Institute of Medical Imaging, 108 Fenglin Road, 200032 Shanghai, China; 4 Department of Radiology, Qilu Hospital of Shandong University, 107 Wenhuaxi Road, 250012 Jinan, Shandong, China; 5 MR Research Collaboration Team, Siemens Healthineers Ltd., 399 Haiyang West Road, 200126 Shanghai, China

**Keywords:** Radiomics, Tomography, X-ray computed, Gastrointestinal stromal tumors, Receptor, Platelet-derived growth factor alpha, Tyrosine kinase inhibitors

## Abstract

**Background:**

Gastrointestinal stromal tumors (GISTs) are the most common mesenchymal tumors of the gastrointestinal tract. Recent advent of tyrosine kinase inhibitors (TKIs) has significantly improved the prognosis of GIST patients. However, responses to TKI therapy can vary depending on the specific gene mutation. D842V, which is the most common mutation in platelet-derived growth factor receptor alpha exon 18, shows no response to imatinib and sunitinib. Radiomics features based on venous-phase contrast-enhanced computed tomography (CECT) have shown potential in non-invasive prediction of GIST genotypes. This study sought to determine whether radiomics features could help distinguish GISTs with D842V mutations.

**Methods:**

A total of 872 pathologically confirmed GIST patients with CECT data available from three independent centers were included and divided into the training cohort ($n = 487$) and the external validation cohort ($n = 385$). Clinical features including age, sex, tumor size and location were collected. Radiomics features on the largest axial image of venous-phase CECT were analyzed and a total of two radiomics features were selected after feature selection. Random forest models trained on non-radiomics features only (the non-radiomics model) and on both non-radiomics and radiomics features (the combined model) were compared.

**Results:**

The combined model showed better average precision (0.250 vs. 0.102, *p* = 0.039) and F1 score (0.253 vs. 0.155, *p* = 0.012) than the non-radiomics model. There was no significant difference in ROC-AUC (0.728 vs. 0.737, *p* = 0.836) and geometric mean (0.737 vs. 0.681, *p* = 0.352).

**Conclusions:**

This study demonstrated the potential of radiomics features based on venous-phase CECT images to identify D842V mutation in GISTs. Our model may provide an alternative approach to guide TKI therapy for patients inaccessible to sequence variant testing, potentially improving treatment outcomes for GIST patients especially in resource-limited settings.

**Supplementary Information:**

The online version contains supplementary material available at 10.1186/s40644-024-00815-3.

## Background

Gastrointestinal stromal tumors (GISTs) are the most common type of mesenchymal tumors in the gastrointestinal tract. They originate from the interstitial cells of Cajal or their stem cell-like precursors. The majority of GISTs are driven by activating mutations in proto-oncogene c-KIT (KIT) in approximately 80% of cases, and platelet-derived growth factor receptor alpha (PDGFRA) receptor tyrosine kinases in 5–10% of cases [1, 2]. The advent of tyrosine kinase inhibitors (TKIs), such as imatinib mesylate and sunitinib malate, which specifically target these mutations, has significantly improved the prognosis of GIST patients [3, 4].

Response to TKI therapy can vary depending on the specific gene mutation in GISTs. Early clinical research has reported that 83.5% of GISTs with mutations in KIT exon 11 and 47.8% with mutations in exon 9 exhibit a partial response to imatinib [5]. Conversely, the predominant PDGFRA mutant isoform, D842V, which accounts for approximately 60% of all PDGFRA mutant GISTs, is completely resistant to imatinib and sunitinib [2, 5, 6]. Patients with D842V-mutant GISTs have a short progression-free and overall survival, necessitating alternative TKIs and treatment strategies [7].

Advances in TKI therapy have emphasized the importance of gene mutation screening and routine imaging follow-up. However, sequence variant testing is costly and may not be affordable or accessible for some patients, especially in low- and middle-income countries, limiting personalized TKI treatment options for these patients [8]. Radiomics is an emerging field that involves the extraction of quantitative features from medical images to predict clinical outcomes and guide treatment decisions. Previous research has demonstrated the potential of radiomics to predict the genotype of GISTs based on contrast-enhanced computed tomography (CECT) images [9–11]. These results indicate that radiomics features have the potential to capture important biological information from GISTs, enabling non-invasive prediction of their genotype and guiding subsequent treatment decisions.

The aim of this multi-center study is to identify radiomics features from computed tomography (CT) images that can reliably predict the presence of D842V-mutant GISTs. By achieving this, we aim to develop a non-invasive, cost-effective, and accessible radiomics model for predicting D842V mutation status, thereby facilitating personalized treatment approaches for GIST patients.

## Methods

### Subjects

**Fig. 1:**
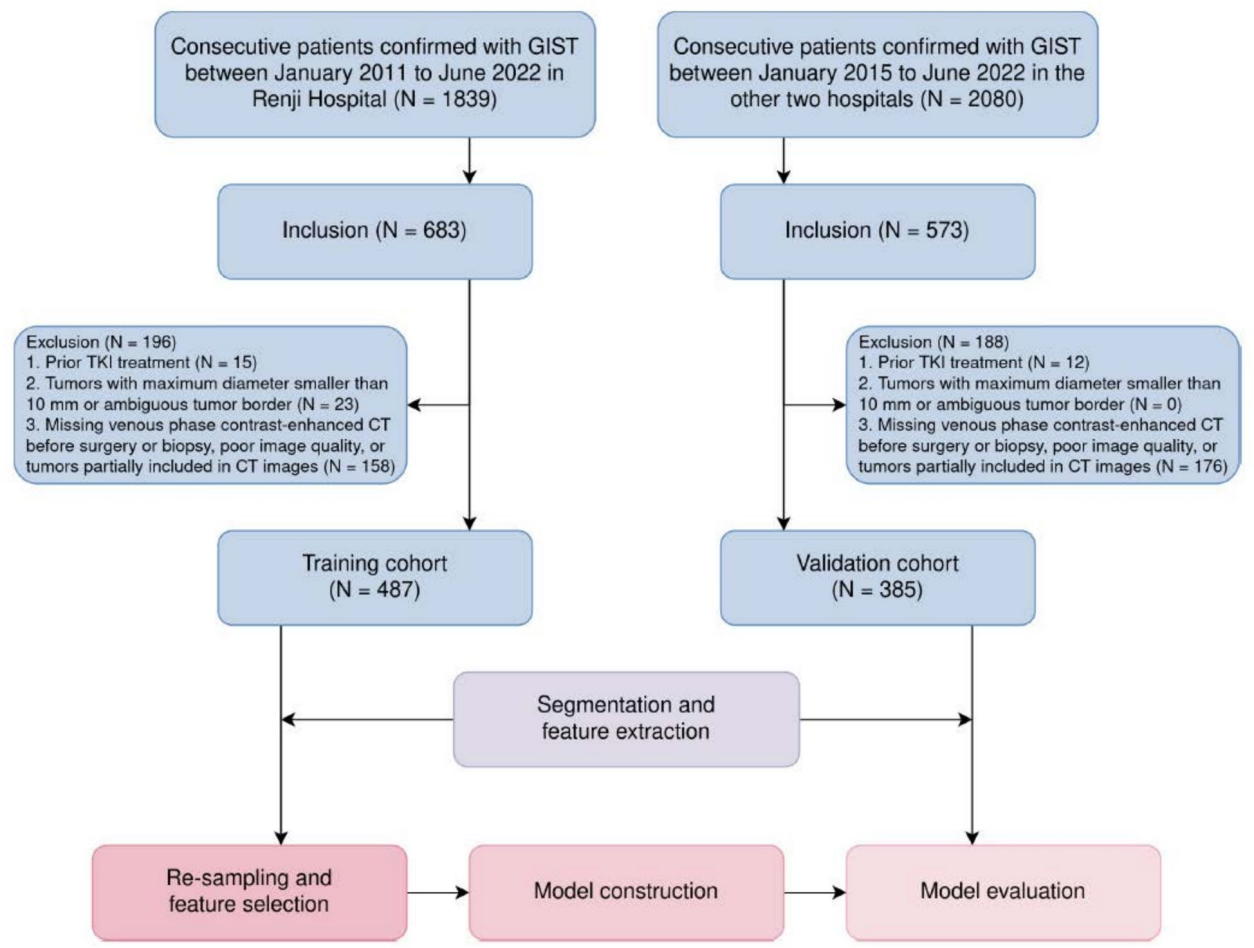
Patient recruitment and study workflow. CT, computed tomography; GIST, gastrointestinal stromal tumors; TKI, tyrosine kinase inhibitors

The institutional review board approved the study protocol (KY2023-002-B), and the study was conducted in accordance with ethical principles of the 1975 Declaration of Helsinki and subsequent revisions. Informed consent from the patients was waived due to the retrospective study design. This retrospective multi-center study enrolled 487 consecutive GIST patients diagnosed with pathologically confirmed GIST between January 2011 and June 2022 in our center, and 385 GIST patients from two other centers between January 2015 and June 2022 (Fig. 1). The inclusion criteria were as follows: (1) Tumors were pathologically confirmed as GIST by fine needle aspiration or surgery; (2) Molecular testing results were available. The exclusion criteria were as follows: (1) Patients with prior TKI treatment; (2) Tumors with its maximum diameter less than 10 mm or ambiguous tumor border on CT images; (3) Missing venous phase CECT before surgery or biopsy, poor image quality, or tumors partially included on CT images. This study adheres to the Image Biomarker Standardisation Initiative (IBSI) guidelines for the standardization of image acquisition and analysis.

### CT image acquisition

All subjects underwent a CECT scan with the scanning and reconstruction parameters used in daily clinical practice. A non-contrast-enhanced CT scan was first obtained before the administration of iodine contrast. Then, the iodine contrast agent was administered at a dose of 1.5 mL/kg of the patient’s weight at a flow rate of 2.5–3.0 mL/s. Arterial (30 - 40 seconds after injection) and venous (70 - 90 seconds after injection) phases were obtained for each patient. Detailed CT protocols are available in Additional file 1.

### CT image segmentation

A radiologist with five years of experience in gastrointestinal imaging (ZX) performed CT image segmentation on the venous phase images. Three months later, the same radiologist (ZX) re-segmented a randomly selected subset of 40 patients, and another radiologist (WZ), with seven years of experience, segmented the images once on the same subset. All CT images were imported into 3D Slicer (version 5.0.3, available at https://www.slicer.org/) for delineation. A 2D region of interest (ROI) of the tumor lesion was delineated on the largest axial slice. CT images of other contrast-enhanced phases and non-contrast-enhanced acquisition were accessible to the radiologists for reference. The radiologists were blind to the pathology results during the segmentation process. Subsequently, all segmented volumes were resampled to a resolution of 1mm * 1mm using linear interpolation, and the images were discretized with a bin width of 25 prior to the extraction of radiomics features.

### Feature selection and model development

Four non-radiomics features, including age, sex, tumor diameter and tumor location were collected from electronic medical records (age and sex), CT images (tumor diameter) and surgical records (tumor location) respectively. These features were either general demographics or employed in the latest guidelines for risk stratification of GISTs. A total of 1023 radiomics features were extracted from the ROI of each CT image with and without filters. These features included first order statistics and textural features obtained from grey-level co-occurrence matrix (GLCM), grey-level dependence matrix (GLDM), grey-level run-length matrix (GLRLM), grey-level size-zone matrix (GLSZM) and neighboring grey tone difference matrix (NGTDM). Features with intra- and inter-observer correlation coefficients greater than 0.75 were selected for model development. Details of the radiomics extraction workflow are provided in Additional file 1.

**Fig. 2:**
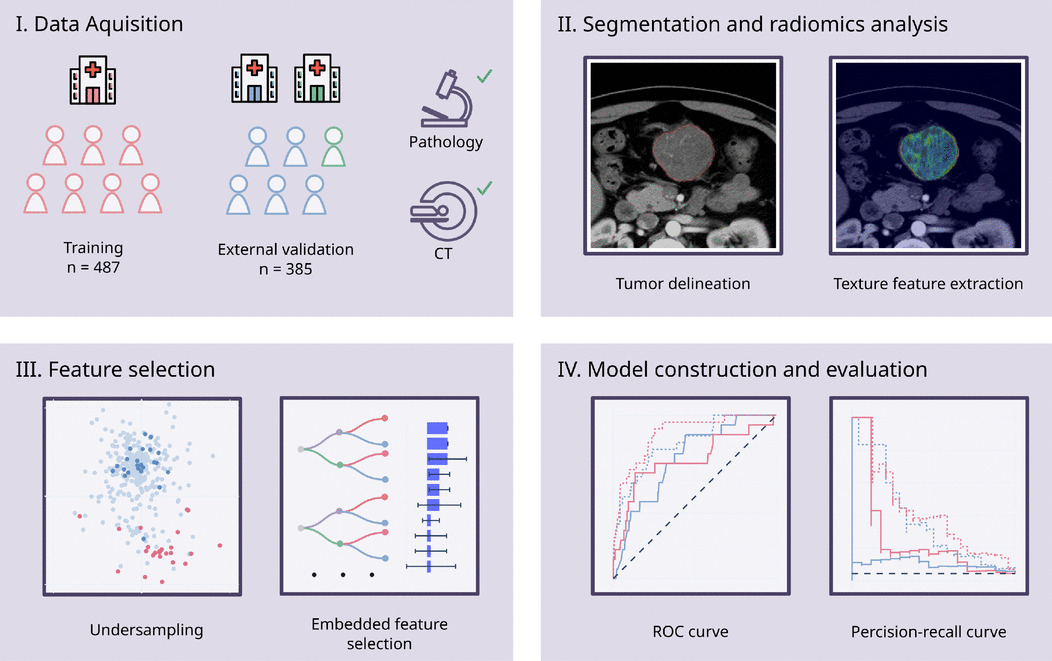
The study design. CT, computed tomography; ROC, relative operating characteristic. Some icons designed by OpenMoji (https://github.com/hfg-gmuend/openmoji) were used under the terms of Creative Commons Share Alike License 4.0 (CC BY-SA 4.0), with modifications to suit our figure requirements

To address the issue of data imbalance, random under-sampling was applied to the D842V wildtype group, and the guided regularized random forest algorithm was employed as an approach of dimensionality reduction [12]. The guided regularized random forest algorithm uses the importance scores from a pretrained random forest model, and adds a penalty on the features used for splitting if their information gain is similar to features used at previous splits. To address the issue of overfitting, a regularization coefficient of 0.5 was employed, and the maximum depth of decision trees was limited to 2. A minimal subset of radiomics features was selected, and a random forest model was built on these radiomics features in conjunction with non-radiomics features. Prior weights were balanced before training the random forest model on the entire training cohort. For comparison, two extra prediction models were developed using the same parameters. One was built using only non-radiomics features, and the other was built using all radiomics and non-radiomics features except tumor location. The predictive capabilities of these models were then evaluated on the entire validation dataset. The radiomics features were extracted using PyRadiomics, version 3.0.1 in Python software, version 3.10.8 (Python Software Foundation), which is compliant to IBSI definitions. The overall study design is illustrated in Fig. 2.

### Statistical analysis

Descriptive statistics were presented as frequencies ($n$) and percentages (%) for categorical variables and mean ± standard deviation for continuous variables. Comparisons of categorical variables between different groups were performed using Fisher’s exact test or Pearson’s $\chi ^2$ test with Yate’s correction for continuity. Continuous variables were compared using students’ t test. Bootstrap was performed with 1000 times of resampling to calculate the variance of different metrics of prediction models, and bootstrap test was performed to compare the performance of them between different models. A two-sided $p$ value of $<0.05$ was considered statistically significant. All statistical analyses were performed with Python software, version 3.10.8 (Python Software Foundation).

## Results

From January 2011 to June 2022, a total of 872 patients were retrospectively included in the study. These patients were divided into the internal training cohort ($n = 487$) from one center, and the external validation cohort ($n = 385$) from the other two centers. Baseline characteristics are shown in Table 1. Patients in the training cohort had higher proportion of GISTs with KIT 11 mutation ($p = 0.044$) and lower proportion of those without KIT and PDGFRA mutation ($p < 0.001$). No statistically significant differences in age, sex and tumor location were observed between the training and validation cohorts.

There were 24 patients with D842V-mutant GISTs in the training cohort (4.93%) and 17 patients in the validation cohort (4.42%). The demographic data of the training and validation cohorts, stratified by D842V mutation, were shown in Table 2 The proportion of D842V-mutant GISTs located in the stomach was significantly higher than that of D842V-wildtype GISTs in both the training cohort and the validation cohort($p < 0.001$). A significantly higher proportion of male patients was observed in D842V-mutant GISTs in the validation cohort (*p* = 0.043). The proportion of male patients was also higher in the training cohort, although this difference was not statistically significant (*p* = 0.077).

**Table 1:** Baseline characteristics of patients in the training and validation cohort

	Training cohort ($\varvec{n = 487}$)	Validation cohort ($\varvec{n = 385}$)	*p*
Age (years)	61.32 ± 12.01	60.63 ± 11.02	0.382
Sex			0.089
Female	238 (48.87%)	165 (42.86%)	
Male	249 (51.13%)	220 (57.14%)	
Location			0.201
Stomach	298 (61.19%)	258 (67.01%)	
Duodenum	45 (9.24%)	38 (9.87%)	
Small intestine	100 (20.53%)	63 (16.36%)	
Others	44 (9.03%)	26 (6.75%)	
Gene mutation			
KIT 9	40 (8.21%)	32 (8.31%)	1.000
KIT 11	378 (77.62%)	275 (71.43%)	0.044
KIT 13	13 (2.67%)	10 (2.60%)	1.000
KIT 17	6 (1.23%)	7 (1.82%)	0.669
PDGFRA 12	6 (1.23%)	0 (0.00%)	0.037
PDGFRA 18	28 (5.75%)	23 (5.97%)	1.000
Wildtype	22 (4.52%)	46 (11.95%)	< 0.001

**Table 2:** Baseline characteristics of patients in the training and validation cohort stratified by D842V mutation

	Training cohort			Validation cohort		
	D842V-mutant ($\varvec{n = 24}$)	D842V-wildtype ($\varvec{n = 463}$)	*p*	D842V-mutant ($\varvec{n = 17}$)	D842V-wildtype ($\varvec{n = 368}$)	*p*
Age (years)	59.79 ± 11.91	61.40 ± 12.02	0.523	63.53 ± 9.72	60.50 ± 11.07	0.268
Sex			0.077			0.043
Female	7 (29.17%)	231 (49.89%)		3 (17.65%)	162 (44.02%)	
Male	17 (70.83%)	232 (50.11%)		14 (82.35%)	206 (55.98%)	
Location			< 0.001			< 0.001
Stomach	21 (87.50%)	277 (59.83%)		16 (94.12%)	242 (65.76%)	
Duodenum	1 (4.17%)	44 (9.50%)		1 (5.88%)	37 (10.05%)	
Small intestine	0 (0.00%)	100 (21.60%)		0 (0.00%)	63 (17.12%)	
Others	2 (8.33%)	42 (9.07%)		0 (0.00%)	26 (7.07%)	

**Fig. 3:**
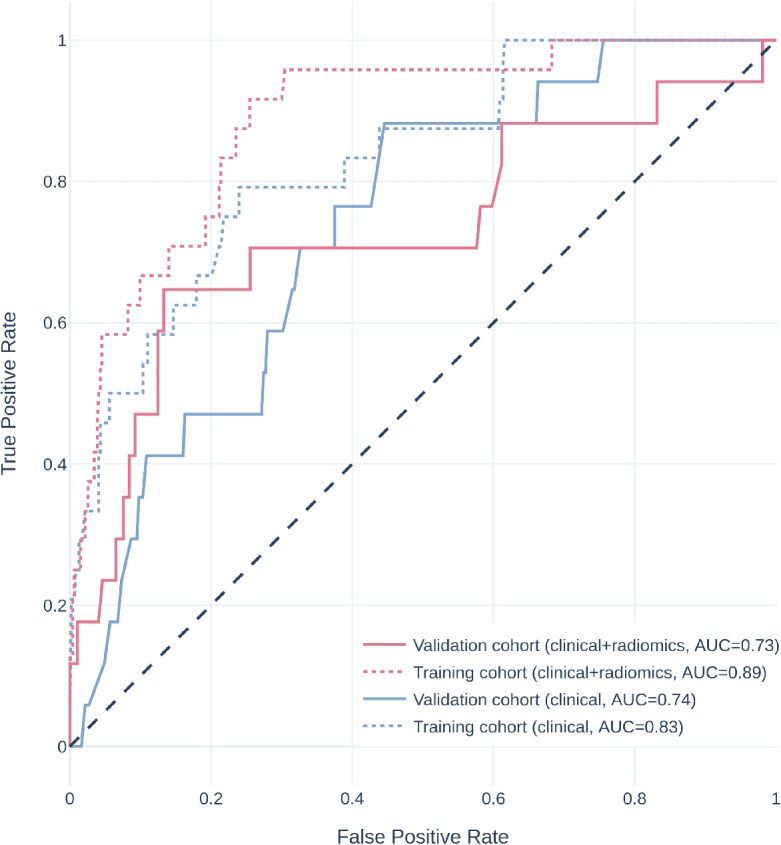
Relative operating characteristic curve of different models. AUC, area under the relative operating characteristic curve

**Fig. 4:**
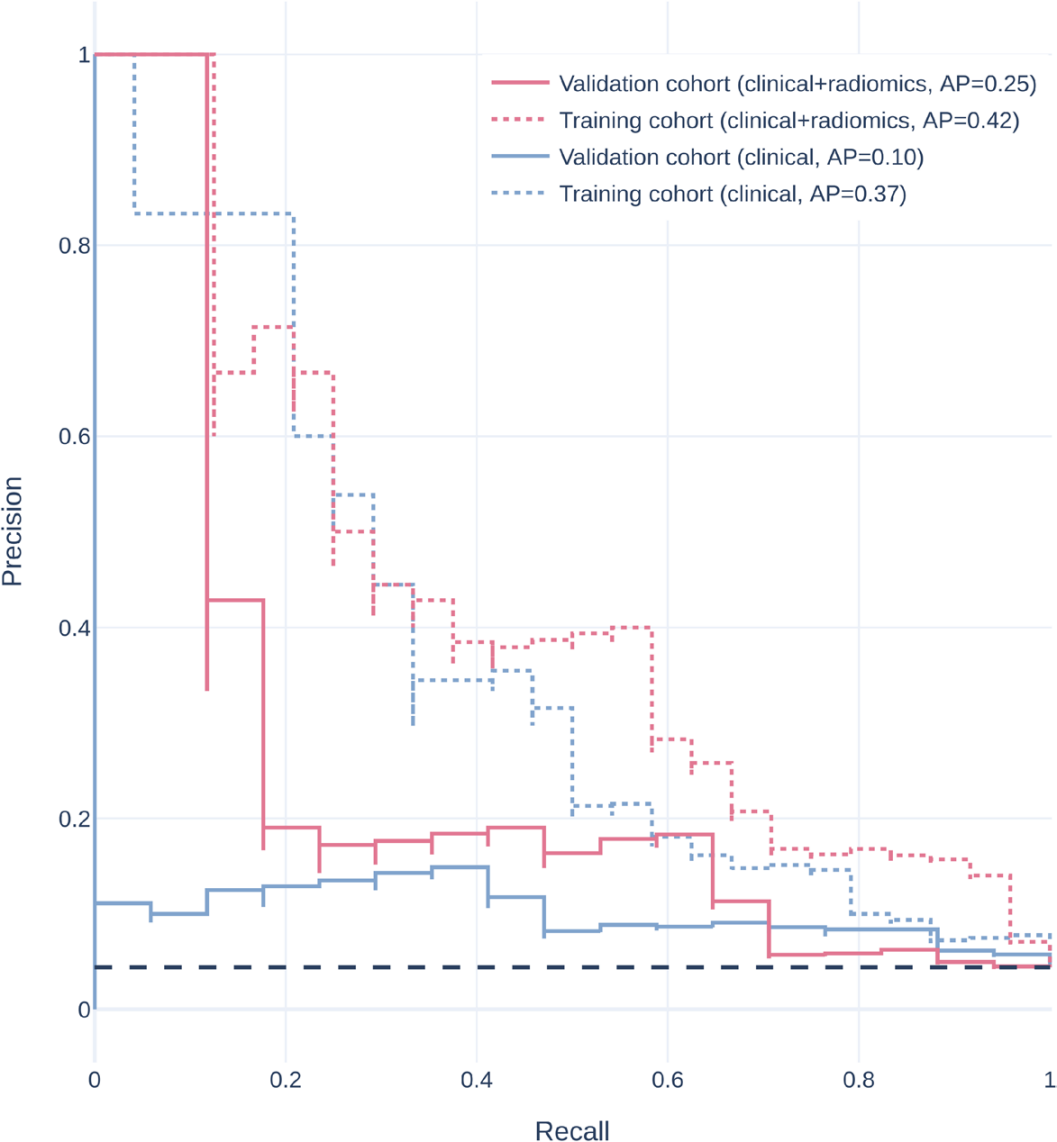
Precision-recall curve of different models. AP, average precision

**Fig. 5:**
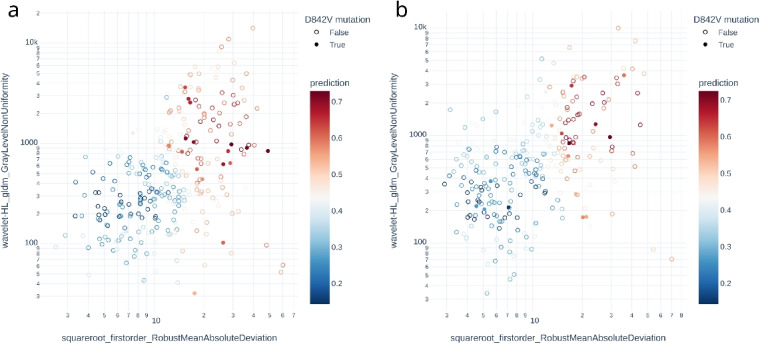
Scatter plot of radiomics features and prediction of the combined model in gastric GISTs. The combined model yields good performance on both the training cohort (**a**) and the validation cohort (**b**)

A total of 601 radiomics features with intra- and inter-observer correlation coefficients greater than 0.75 were selected for model development. After feature selection, two radiomics features, namely wavelet-HL filtered GLDM GrayLevelNonUniformity and squareroot filtered RobustMeanAbsoluteDeviation, were selected for use in training prediction models. The predictive performance of the model trained on non-radiomics features alone (the non-radiomics model) and on both non-radiomics and radiomics features (the combined model) were shown in Table 3. Relative operating characteristic (ROC) curve and Precision-recall curve of the models on both the training and the validation cohort were shown in Figs. 3 and 4. The combined model showed better average precision (0.250 vs. 0.102, $p = 0.039$) and F1 score (0.253 vs. 0.155, $p = 0.012$) than the non-radiomics model. There was no statistical significance between the two models in area under the relative operating characteristic curve (ROC-AUC) and geometric mean. The predictions of different models were illustrated in Fig. 5. Results on the model trained on all radiomics and non-radiomics features except tumor location were presented in Additional file 1.

**Table 3:** Diagnostic performance of non-radiomics and combined models

	Clinical model		Clinical+radiomics model		
	value	95% CI	value	95% CI	*p*
AP	0.102	[0.045, 0.192]	0.250	[0.097, 0.486]	0.039
G-Mean	0.681	[0.542, 0.776]	0.737	[0.574, 0.860]	0.352
F1	0.155	[0.088, 0.254]	0.253	[0.143, 0.400]	0.012
ROC-AUC	0.737	[0.609, 0.833]	0.728	[0.516, 0.861]	0.836

*AP* average precision, *G-Mean* geometric mean, *ROC-AUC* area under the relative operating characteristic curve, *CI* confidence interval

## Discussion

In this work, we explored the potential of CT imaging features to detect PDGFRA exon 18 D842V mutation. We developed a prediction model incorporating non-radiomics variables and radiomics features from venous phase CECT, which showed robust performance in predicting D842V-mutant GISTs. Although the combined model had a similar ROC-AUC compared to the non-radiomics model, the combined model had a significantly higher average precision in the precision-recall curve (Fig. 4), which is considered more robust in imbalanced settings. In addition, the combined model showed significantly higher F1 score. The results of this study promoted that D842V-mutant GISTs may exhibit distinct imaging phenotypes that can help to differentiate them from D842V-wildtype GISTs. To our best knowledge, this is the first study to leverage imaging features to predict D842V-mutant GISTs. This is particularly important as our findings could enable early and cost-effective identification of a specific primarily imatinib-resistant genotype of GISTs.

The response of GISTs to TKI therapy is largely dependent on the location of gene mutation, thus sequence variant testing is always recommended for GIST patients [5]. Despite the benefits of genomic sequencing, disparities based on race, ethnicity and geographic region can limit patients’ access to optimal treatments, potentially exacerbating health-care associated impoverishment [8, 13, 14]. A recent study revealed that GIST patients in low- and middle-income countries, who may not have access to sequence variant testing, had treatment outcomes comparable to those reported in high-income countries after first-line imatinib and second-line sunitinib therapies [15]. One possible explanation for these favorable results was that GISTs with KIT mutation, which was the predominant gene mutation, were mostly sensitive to imatinib or sunitinib treatment. However, our data indicated that at least 5% of patients had D842V mutation with primary resistance to imatinib. Prescribing TKI therapy without detailed sequence variant testing could lead to the futility of ineffective treatments and the unnecessary imposition of adverse effects on these patients. Therefore, early identification of these patients in a more accessible approach is of significant clinical importance and is urgently needed.

Previous research have shown that GISTs with D842V mutation were predominantly found in the stomach and omentum [16, 17]. Our study confirmed these findings, and we additionally found a male predominance in these cases. Nevertheless, our prediction model based on non-radiomics features alone cannot adequately detect D842V mutations, probably due to the relatively low prevalence of this genotype. After removal of tumor location, we found a decrease in all performance metrics. However, the model remained effective with both ROC-AUC and average precision above chance level, suggesting that our combined model is not dependent on tumor location alone, and that radiomics features have a significant contribution in predicting D842V mutation.

Imaging-based prediction models have been increasingly recognized for their utility in predicting specific gene mutation variants in GISTs with the help of radiomics. Xu et al. reported that CECT texture features, particularly the standard deviation of tumor intensity, could discriminate GISTs without KIT exon 11 mutation from those with KIT exon 11 mutation [18]. Wei et al. discovered that CECT-based radiomics features and clinical features, including extra-gastric location and distant metastasis, could effectively predict KIT exon 9 mutation [11]. In our previous work, we demonstrated that imaging-based quantitative features could distinguish between different genotypes of KIT exon 11 mutations, especially deletions involving codons 557/558 [9]. We also found that GISTs may exhibit specific imaging patterns at different phases of CECT images in predicting Ki-67 proliferation index [19]. These studies highlighted the potential of imaging features to discriminate between various gene mutations in GISTs. Consistent with these studies, our combined model showed a significant improvement in both average precision and F1 score over the non-radiomics model. Our study further demonstrated that radiomics features were capable of predicting PDGFRA exon 18 D842V mutations in GISTs. Given the relative availability of CT imaging, we believe the application of our prediction model could assist decision making in TKI treatment and the subsequent patient monitoring, particularly in populations with limited access to sequence variant testing.

### Limitations

Our study had inevitably some limitations. The retrospective study design introduced potential selection bias, which may impact the generalizability of the findings. The radiomics features were examined from the largest axial slice of the tumor in the venous phase, which may not provide a comprehensive representation of the tumor. Nevertheless, we believe the impact is limited given that comparable performance has been achieved in previous studies between two- and three-dimensional radiomics features, as well as across different contrast-enhanced phases in gastrointestinal tumors [20, 21]. Although D842V mutation is the most frequently identified imatinib-resistant genotype according to prior research, patients with D842V mutant GISTs represent a minor proportion of the overall GIST population. The imbalanced patient cohort, coupled with a high-dimensional dataset, posed challenges in achieving a representative sample size and a balanced distribution of mutations across patient subgroups. To mitigate these challenges, we collected multi-center data to maximize the number of samples available for analysis. Furthermore, we employed random under-sampling in combination with random forest based feature selection to minimize the risk of overfitting. Random under-sampling was chosen considering that the more popular over-sampling methods could potentially increase the likelihood of overfitting in the induction process [22]. The guided regularized random forest algorithm was selected for its reputation as a common embedded feature selection approach that had been successfully applied in high-dimensional and imbalanced datasets, similar to our settings [12, 23]. Despite these methodological strategies, these limitations remain and should be carefully considered when interpreting the results. Finally, the high-order radiomics features used in our model are complicated and difficult to explain, which restrict the interpretability of our findings. Future research is required to confirm these results in clinical settings.

## Conclusions

In conclusion, our study successfully developed a prediction model that integrates non-radiomics variables with radiomics features from venous phase CECT and demonstrated its efficacy in identifying D842V-mutant GISTs. The integration of imaging features into the prediction model significantly improved its performance, which could facilitate the early, cost-effective detection of a genotype resistant to current first- and second-line TKI therapy. Our model may provide an alternative approach to guide TKI therapy for patients inaccessible to sequence variant testing, potentially improving treatment outcomes for GIST patients especially in resource-limited settings. Further research and validation studies will be necessary to refine the model and to evaluate its performance in a clinical setting.

## Supplementary Information

Supplementary Material 1.

## Data Availability

The source code and interactive figures for our paper was publicly available at https://github.com/heyrict/gist-d842v-open. Data in our paper was available upon reasonable request to the corresponding authors.

## Abbreviations

AP: Average precision
CECT: Contrast-enhanced computed tomography
CI: Confidence interval
CT: Computed tomography
G-Mean: Geometric mean
GIST: Gastrointestinal stromal tumor
GLCM: Grey-level co-occurrence matrix
GLDM: Gray-level dependence matrix
GLRLM: Grey-level run-length matrix
GLSZM: Grey-level size-zone matrix
IBSI: Image Biomarker Standardisation Initiative
KIT: Proto-oncogene c-KIT
NGTDM: Neighbouring gray tone difference matrix
PDGFRA: Platelet-derived growth factor receptor alpha
ROC: Relative operating characteristic
ROC-AUC: Area under the relative operating characteristic curve
ROI: Region of interest
TKI: Tyrosine kinase inhibitor
