# Supplementary material for: Identification of D842V mutation in gastrointestinal stromal tumors based on CT radiomics: a multi-center study
Source: Cancer Imaging. 2024 Dec 20;24:169. doi: 10.1186/s40644-024-00815-3 (PMC11662607; doi:10.1186/s40644-024-00815-3)
Supplement: Supplementary file 1 — Supplementary Material 1. [file 40644_2024_815_MOESM1_ESM.pdf]

Additional file for “Identification of D842V mutation in gastrointestinal stromal tumors based on CT radiomics: A multi-center study”

1 Protocols of CT scanners in different centers

Table A1: Protocols of CT scanners in different centers

Parameters	Center 1	Center 2	Center 3
CT version	Spectral CT (Discovery CT750 HD scanner, GE Healthcare, USA)	Spectral CT (Aquilion One TSX-301A, TOSHIBA, Japan)	Philips Brilliance 256-slice helical CT scanner (Brilliance ICT, Philips, Netherlands)
CT tube voltage	120 kVp	120 kVp	120 kVp
CT tube current	220 mA	60 mA	160–600 mA
CT rotation time	0.6 s	0.6 s	0.5 s
CT detector collimation	64×0.625 mm	64×0.5 mm	128×0.625 mm
Contrast agent type	Iopamidol, Iopamiro, Bracco Sine, Shanghai, China	Iopamiro, Bracco Sine, Shanghai, China	Iopromide, Bayer Medical, Berlin, Germany
Contrast agent concentration	370 mgI/ml	300 mgI/ml	350 mg I/mL
Contrast agent dosage	1.5 ml/kg	1.5 ml/kg	1.5 ml/kg
Contrast agent flow rate	3.0 ml/s	3.0 ml/s	2.5–3.0 mL/s
Image matrix	512×512	512×512	512×512
Field of view	500×500 mm	500×500 mm	500×500 mm
Reconstruction image thickness	1 mm	1 mm	1 mm

2 Detailed radiomics extraction workflow

Gastrointestinal stromal tumor (GIST) lesions were segmented on the largest axial slice with 3D Slicer (version 5.0.3, https://www.slicer.org/). The computed tomography (CT) images were first exported from the picture archiving and communication system (PACS) in our hospital, and imported into 3D Slicer as scalar volumes. For consistency, these scalar volumes were exported in NRRD format for later use in radiomics analysis. Radiologists who delineated the GIST lesion had access to non-contrast-enhanced CT images as well as all contrast-enhanced phases if available. Only the venous phase of the contrast-enhanced CT was delineated. Pathology results were also available to radiologists for reference about the location of GIST. Radiologists were instructed to ensure that the segmentations were strictly contained in the tumor, and may or may not include the partial volume effects in the tumor margin. Air, or other gastric contents should be carefully avoided or removed after delineation. If there were abdominal dissemination, delineation of the tumor as indicated by the pathology results was preferred, otherwise the largest slice of the largest contiguous mass was delineated. No specific instructions on delineation methods was enforced, i.e. manual or semi-auto delineation were both allowed for radiologists as long as the segmentation followed the above restrictions.

Delineation was initially performed by one radiologist (ZX) with 5 years of experience in gastrointestinal imaging. Three months later, the image segmentation process was repeated by the same radiologist (ZX) on a randomly selected subset of 40 patients as the intra-observer correlation dataset. Another radiologist (WZ) with 7 years of radiology experience performed image segmentation once on the same subset as the inter-observer correlation dataset. Segmentations of GISTs were exported directly from Slicer as NRRD format (.seg.nrrd).

Scalar volumes of CT images and segmentations were read as SimpleITK (version 2.3.1) images. The segmentation geometry was corrected and data were resampled using PyRadiomics (version 3.0.1). All segmented volumes were resampled to 1mm * 1mm * 5mm with linear interpolation, and the image was discretized with a bin width of 25 before the extraction of radiomics features. Segmentations were resampled to the same resolution with nearest neighbor interpolation.

In addition to the original image, filters including wavelet (in 4 dimensions, HL, HH, LH, LL), square, squareroot, logarithm, exponential, gradient and local binary patten (applied in 2D) were also performed before feature extraction. Features of first order statistics, shape, grey-level co-occurrence matrix (GLCM), gray-level dependence matrix (GLDM), grey-level run-length matrix (GLRLM), grey-level size-zone matrix (GLSZM) and neighbouring gray tone difference matrix (NGTDM), were extracted using PyRadiomics. The default texture paramters from PyRadiomics were used for feature extraction. Explicitly, GLCM was computed using symmetric co-occurrence matrices with a distance of 1. GLSZM was computed with a linkage distance of 1. NGTDM was computed with a neighborhood distance and distance norm of 1.

A total of 978 radiomics features were extracted. Among them, 702 features and 646 features had inter- and intra-observer correlation coefficient greater than 0.75, respectively. A total of 601 features with both inter- and intra-observer correlation coefficient greater than 0.75 were selected for feature selection.

3 Results on the model without tumor location

Table A2: Diagnostic performance of models with or without tumor location.

	model without location	(95% CI)	model with location	(95% CI)	p
AP	0.176	[0.058, 0.388]	0.250	[0.097, 0.486]	0.056
G-Mean	0.598	[0.404, 0.781]	0.737	[0.574, 0.860]	0.064
F1	0.194	[0.087, 0.346]	0.253	[0.143, 0.400]	0.203
ROC-AUC	0.654	[0.456, 0.806]	0.728	[0.516, 0.861]	0.004

AP, average precision; PR-AUC, area under the precision recall curve; G-Mean, geometric mean; ROC-AUC, area under the relative operating characteristic curve; CI, confidence interval.

Detailed comparison between this model trained on all features except tumor location (model without location) and the combined model (model with location) is provided in Table A2. After removal of tumor location, there was a decline in all performance metrics. The ROC-AUC of the model without tumor location was significantly lower than the combined model ($p = 0.004$).

References
